# DNA mismatch repair protein Mlh1 is required for tetravalent chromium intermediate-induced DNA damage

**DOI:** 10.18632/oncotarget.20150

**Published:** 2017-08-10

**Authors:** Timothy P. Wakeman, Aimin Yang, Naresh S. Dalal, Rebecca J. Boohaker, Qinghua Zeng, Qiang Ding, Bo Xu

**Affiliations:** ^1^ Department of Biochemistry and Molecular Biology, LSU Health Sciences Center, New Orleans, LA, USA; ^2^ Department of Genetics, LSU Health Sciences Center, New Orleans, LA, USA; ^3^ Department of Nuclear Medicine, First Affiliated Hospital, Xi’an Jiaotong University, Xi’an, China; ^4^ Department of Chemistry and Biochemistry, Florida State University, Tallahassee, FL, USA; ^5^ Department of Oncology, The Southern Research Institute, Birmingham, AL, USA; ^6^ Department of Cell, Developmental, and Integrative Biology, University of Alabama at Birmingham, Birmingham, AL, USA

**Keywords:** chromium exposure, Cr intermediates, carcinogenicity, DNA double-strand break, mismatch repair

## Abstract

Hexavalent chromium (Cr[VI]) is associated with occupational lung cancer and poses a significant public health concern. When exposed to Cr[VI], cells rapidly internalize this compound and metabolize it to Cr[III]. Byproducts of Cr[VI] metabolism include unstable Cr[V] and Cr[IV] intermediates that are believed to be directly responsible for the genotoxicity and carcinogenicity caused by Cr[VI] exposure; however, the carcinogenic potential of the Cr intermediates and the mechanisms of Cr-induced carcinogenesis remain to be further defined. Utilizing synthetic Cr[IV] and Cr[V] compounds, we demonstrate here that Cr[IV] or Cr[V] exposure induces DNA double-strand breaks; however, of the two compounds, mammalian cells only respond to Cr[V]-induced DNA damage. Exposure to Cr[V], but not Cr[IV], results in initiation of cell cycle checkpoints and activates the ATM kinase, a critical regulator of the DNA damage response. Furthermore, cells exposed to Cr[IV] have significantly increased mutation frequencies in the HPRT gene compared to cells exposed to Cr[V], indicating that Cr[IV] possesses a higher mutagenic potential than Cr[V]. We also find that MLH1, a critical mismatch repair (MMR) protein, is required for activation of the G2/M cell cycle checkpoint in response to Cr[VI] exposure and to limit Cr-induced mutagenesis. Our results provide evidence for Cr[IV] as the ultimate mutagenic intermediate produced during Cr[VI] metabolism and indicate that functional MMR is crucial in the cellular response to chromium exposure.

## INTRODUCTION

Chromium compounds exist in nature almost exclusively in the trivalent (Cr [III]) or hexavalent (Cr [VI]) state, with the relatively non-reactive Cr[III] being predominant. Cr[III] is an essential nutrient required for proper insulin function and is harmless even at moderately high levels. Cr[VI] compounds are common industrial waste products, many of which are water soluble and can readily pollute the environment through direct release into lakes and streams. These compounds are also released into the air, exposing workers to high levels of Cr[VI]. Cr[VI] compounds are known human carcinogens, and workers exposed to these compounds exhibit high incidences of nasal, esophageal, and lung cancers. Both Cr[III] and Cr[VI] compounds enter the body through respiratory and gastric epithelia, but only Cr[VI] readily enters cells. Cr[VI] is transported into cells via the sulfate anion transporter where it is reduced ultimately to Cr[III] by glutathione and ascorbate. Highly reactive pentavalent (Cr[V]) and tetravalent (Cr[IV]) intermediates are generated during this reduction. These intermediates can cause intracellular damage by reacting with DNA and proteins. Additionally, reduction of these intermediates results in the formation of oxygen radicals that can interact with DNA causing a wide variety of damage [1–3].

DNA damage resulting from exposure to Cr[VI] includes DNA intrastrand crosslinks, Cr-DNA adducts, and DNA single-strand breaks [4]. Exposure to Cr[VI] also results in the formation of DNA double-strand breaks, which are highly genotoxic lesions capable of inducing mutations [5]. Cr[VI] cannot directly interact with DNA, and studies indicate that it ceases to be genotoxic when experimental conditions do not favor reduction [6]. It is therefore likely that aspects of the reductive process contribute to Cr[VI]-induced genotoxicity and mutation. Specifically, metal-induced oxidative stress caused by high-valent chromate intermediates is thought to be the major mechanism responsible for Cr[VI]-induced DNA damage [7, 8]. To study cellular responses and the mutagenic potential of these intermediates, we utilized synthetic chromium intermediate compounds that have previously been discussed by Ramsey and Dalal and are determined to be suitable for mimicking endogenously produced Cr[IV] and Cr[V] intermediates [9].

Cr[VI] exposure activates several cellular stress responses, including cell cycle checkpoints and apoptosis [2]. One of the functions of these stress responses is to limit mutation, either through allowing cells time to sense and repair damaged DNA or causing cells with irreparable DNA damage to be removed from the population. Molecular mechanisms regulating these Cr responses are less known, although DNA damage response regulators are predictably involved in the process. For example, there are evidence that exposure to Cr[VI] activates a major DNA damage response protein, the ataxia-telangiectasia mutated (ATM) protein and induces an ATM-dependent S-phase checkpoint [5, 10]. Activation of ATM is an critical step for an optimal DNA damage response [11].

Here, we demonstrate that exposure to either Cr[IV] or Cr[V] resulted in the formation of DNA double strand breaks; however, only Cr[V] exposure results in the activation of ATM and G2 and S-phase arrest. Furthermore, Cr[IV] exposure results in increased mutation frequency in the HPRT gene. We also demonstrate that mismatch repair deficient cells exhibited defective G2/M checkpoint activation and increased HPRT mutation rates compared to mismatch repair proficient cells in response to Cr[VI] exposure. Our results demonstrate that Cr[IV] fails to activate optimal DNA damage responses and is the ultimate mutagenic Cr-metabolite and that mismatch repair is important in limiting Cr-induced mutation.

## RESULTS

### Exposure to Cr[VI], Cr[V], or Cr[IV] induces DNA double-strand breaks

**Figure 1:**
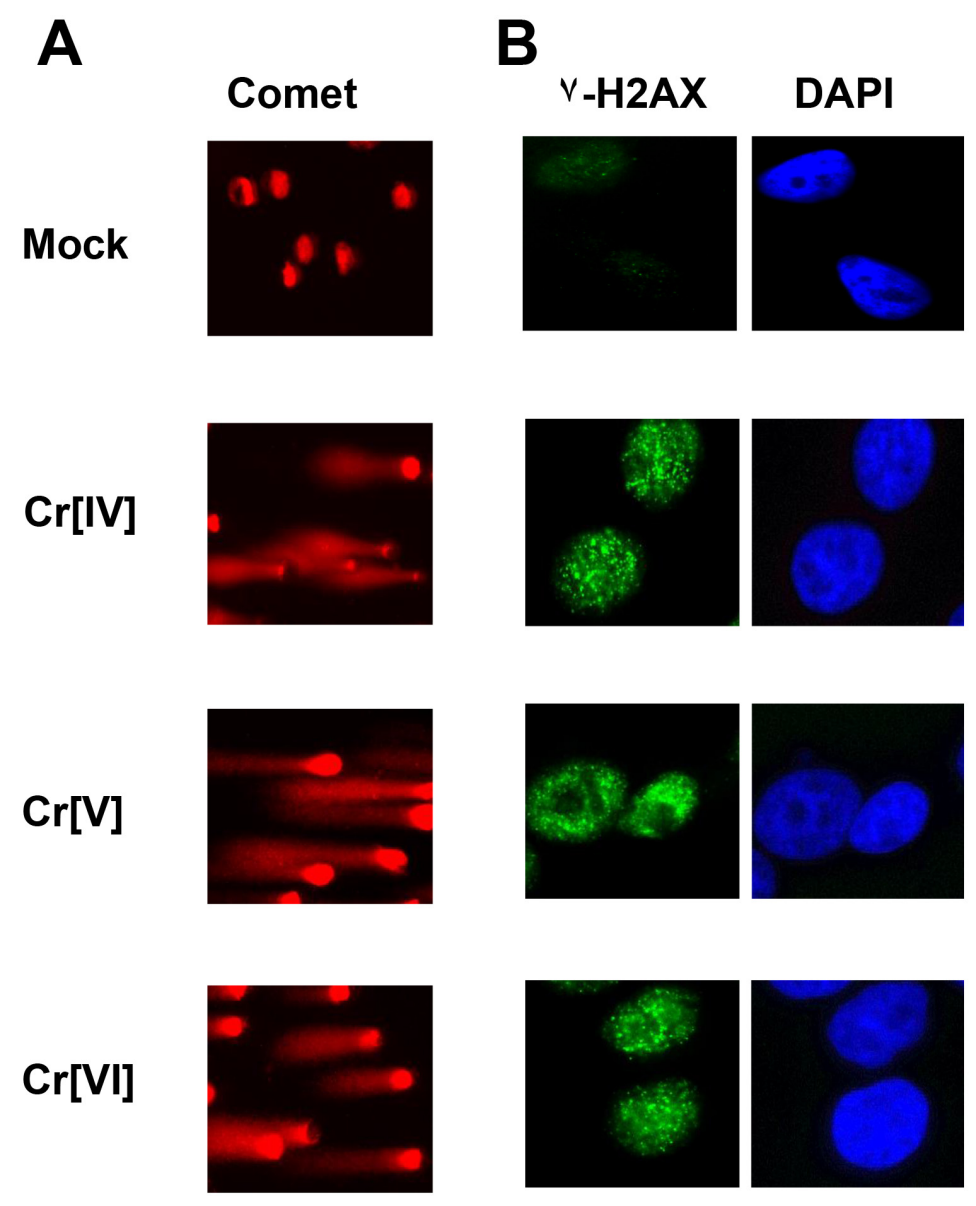
Cellular exposure to Cr[VI], Cr[V], or Cr[IV] results in DNA double-strand breaks HeLa cells were exposed to 10 μM concentrations of Cr[VI], Cr[V], or Cr[IV]. After 30 minutes cells were **(A)** harvested and subjected to comet analysis or **(B)** fixed and stained for γ-H2AX foci formation. Comet tails and γ-H2AX foci are indicative of DNA double-strand breaks.

We previously demonstrated that Cr[VI] exposure results in the formation of DNA double strand breaks [5]; however, Cr[VI] is incapable of directly interacting with the DNA to produce damage. This suggests that intermediates produced during Cr[VI] metabolism are responsible for the formation of these lesions. To investigate the potential for Cr[IV] and Cr[V] to induce DNA double strand breaks, we utilized potassium peroxychromate (K_3_CrO_8_) in the Cr[V] related investigations, and Cr[IV]-diperoxoamine monohydrate complex, (Cr(C_4_H_13_N_3_) (O2)_2_.H_2_0), for the Cr[IV] studies. These compounds were selected because they can be prepared in pure crystalline form, are stable enough for model studies over a few months when stored at 4° C, and have been well characterized by detailed spectroscopic and x-ray structural techniques. The single cell gel electrophoresis (Comet) assay was performed on cells exposed to these compounds. HeLa cells exposed to 10μM concentrations of Cr[VI], Cr[V], or Cr[IV] for 30 minutes displayed comet tail formation, indicating the formation of DNA strand breaks (Figure 1A). To confirm that these were DNA double strand breaks, we stained experimentally treated cells with an antibody specific for γ-H2AX, which is present at sites of DNA double-strand breaks. All experimental groups showed robust γ-H2AX foci formation (Figure 1B), indicating that both Cr[IV] and Cr[V] are capable of inducing DNA double strand breaks. Additionally, we observed no appreciable differences between Cr[IV] and Cr[V] in regards to the number of γ-H2AX foci or size of comet tails induced by exposure to these compounds (data not shown).

### Exposure to Cr[IV] fails to initiate optimal cell cycle checkpoints

**Figure 2:**
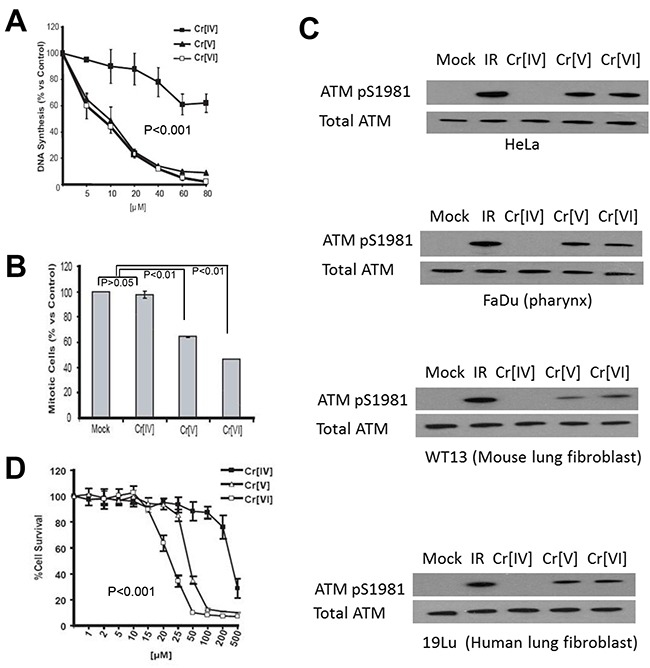
Exposure to Cr[IV] fails to induce an optimal DNA damage response **(A)** HeLa cells were exposed to increasing doses of the Cr compounds for a period of 4 hours. ^3^H-thymidine incorporation was then performed to determine S-phase checkpoint activation, where a reduction in DNA synthesis is indicative of checkpoint activation. **(B)** HeLa cells were exposed to 40μM doses of the Cr compounds for 4 hours, after which they were stained with propidium iodide and phospho-histone H3 antibody to detect mitotic cells. Reduction in mitotic cells indicates G2 checkpoint activation. **(C)** HeLa, FaDu, WT13, and 19Lu cells treated with 10 μM doses of Cr for 4 hours were lysed and analyzed by western blot for ATM activation, indicated by ATM phosphorylation at S1981. **(D)** HeLa cells were exposed to increasing doses of Cr for 24 hours, after which their survival rates were assayed by MTT analysis.

To investigate the cellular response to the genotoxicity of the chromate intermediates, we first examined the initiation of cell cycle checkpoints in response to these compounds. HeLa cells were exposed to doses of the chromium compounds ranging from 5-80 μM for 4 hours. These cells were then subjected to RDS analysis, which measures DNA synthesis by quantifying ^3^H thymidine incorporation after DNA damage to determine activation of the S-phase checkpoint. Following Cr[VI] exposure, HeLa cells exhibit a significant (p<0.001) decrease in DNA synthesis, representing activation of the S-phase cell cycle checkpoint. Similar to Cr[VI], Cr[V] induces a dose-dependent reduction of DNA synthesis (Figure 2A). Interestingly, cells exposed to Cr[IV] displayed little to no inhibition of DNA synthesis (Figure 2A), suggesting that Cr[IV] fails to induce S-phase arrest. To confirm that this phenotype is not S-phase specific, we also examined the ability of the chromate intermediates to induce a G2/M checkpoint using anti-phospho-Histone H3 staining followed by flow cytometric analysis. Similar to the S-phase response, cells exposed to Cr[VI] or Cr[V] showed dramatic (p<0.01) reduction in the number of mitotic cells, indicating G2 checkpoint activation (Figure 2D). However, Cr[IV] exposure resulted in no reduction in the number of mitotic cells, indicating a lack of G2 arrest in response to this compound. It is noted that in addition to the HeLa cell line, we have confirmed these results by testing several other cell lines, including 293T and human primary fibroblast cell lines (data not shown).

### Exposure to Cr[IV] does not activate the ATM kinase

To further investigate if DNA damage response induced by exposure to Cr[IV] is altered, we examined if the ATM kinase, a DNA damage regulator previously reported to be stimulated by Cr[VI], became activated after exposure to chromium intermediates. To test this, HeLa cells were exposed to 10μM doses of the compounds for 4 hours and harvested for Western Blot analysis using an anti-phospho-ATM antibody. Auto-phosphorylation of ATM at Serine 1981 is indicative of activation of the protein [12]. We observed that cells treated with Cr[VI] or Cr[V] showed ATM auto-phosphorylation, but cells exposed to Cr[IV] displayed no ATM phosphorylation (Figure 2C top panel). To confirm our result, We have used additional relevant cell lines, including human head and neck squamous cell carcinoma FaDu (pharynx epithelia), normal human lung fibroblast 19Lu, and mouse lung fibroblast WT13 cells for validation (Figure 2C lower panels). These WB results show the same pattern as that of HeLa. Taken together, we conclude that cellular DNA damage response to Cr[IV], unlike to Cr[V] or Cr[VI], is significantly reduced.

### Cr[IV] exhibits reduced cytotoxicity compared to Cr[VI] or Cr[V]

Since both Cr[V] and Cr[IV] are genotoxins capable of inducing DNA double strand breaks, we decided to examine the cytotoxicity of these compounds. HeLa cells were exposed to doses of Cr[VI], Cr[V], or Cr[IV] ranging from 1-500μM for 24 hours. These cells were then analyzed by MTT analysis to examine cell viability following Cr exposure. We found that exposure to Cr[VI] or Cr[V] induces significant cell death at doses of 50μM or higher (Figure 2D). Ten-fold greater concentrations of Cr[IV] were required to induce a similar amount of cell death, despite the observation that all the Cr compounds exhibit equal genotoxic potential at doses as low as 10μM. These observations indicate that Cr[V] is the primary cytotoxic intermediate produced by Cr[VI] metabolism.

### Cr[IV] causes increased mutation in the HPRT gene compared to Cr[V]

**Figure 3:**
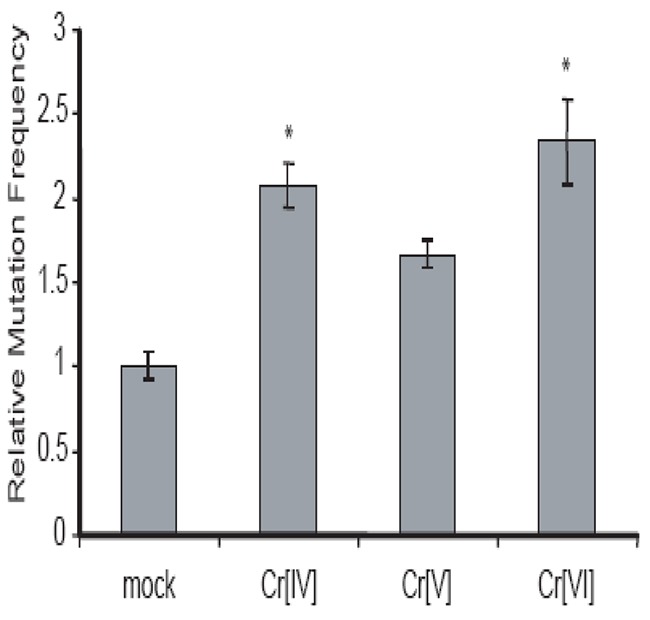
Cr[IV] causes higher rates of mutation in the HPRT gene compared to Cr[V] **(A)** Diagram of the HPRT assay used to determine Cr-induced mutation rates. **(B)** HCT 116 cells were exposed to 10μM doses of the Cr compounds for 24 hours. Analysis of mutation in the HPRT gene was used to obtain Cr-induced mutation frequencies. These frequencies were then normalized relative to spontaneous mutation frequencies (^*^ indicates p-value<.05).

Cr[VI] induces HPRT mutation in human cells [13], but it does not interact directly with DNA. It is therefore likely that intermediates produced during Cr[VI] metabolism are responsible for Cr[VI]-induced mutagenesis. Since both Cr[IV] and Cr[V] are capable of causing DNA DSBs, but cells fail to respond to Cr[IV]-induced DNA damage properly, we hypothesized that Cr[IV] might be the major intermediate contributing to Cr[VI]-induced mutagenesis. To test this hypothesis, we investigated the mutagenic potential of the chromate intermediates by examining their ability to induce mutation in the HPRT gene. The measurement of mutant frequency (Mf) at the HPRT locus is a well-established method for estimating genomic instability [14]. The HPRT mutagenesis assay is based on the fact that cells possessing normal HPRT function will be killed by exposure to the purine analog 6-thioguanine (6-TG), while cells possessing mutations in the HPRT gene are resistant to 6-TG. Human colorectal cancer cell line HCT116 was employed for this assay. Prior to the experiment, cells already possessing mutated HPRT were selected out of the population by culture in medium containing hypoxanthine, aminopterin, and thymidine (HAT medium). The cells were exposed to the Cr compounds and then were selected for loss of function HPRT mutants. After 10 days, the number of colonies was counted for each experimental treatment and statistical analysis was performed to determine the Cr-induced Mf relative to untreated cells. As shown in (Figure 3), each of the three Cr compounds were able to induce an increase in HPRT mutation, but exposure to Cr[IV] or Cr[VI] produced a significant increase (p<.05) in Mf compared to mutation frequencies induced by Cr[V]. These observations indicate that Cr[IV] is the Cr intermediate that contributes to Cr[VI]-induced mutagenesis.

### Mismatch repair is required for G2/M checkpoint activation following Cr[VI] exposure

**Figure 4:**
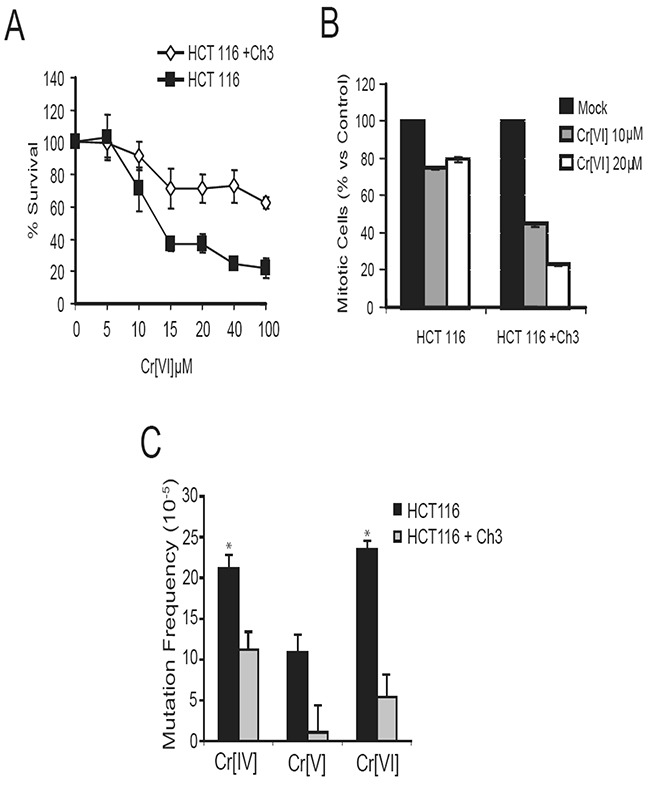
Functional mismatch repair is required for G2/M checkpoint activation and to limit mutation in response to Cr exposure **(A)** HCT116 and HCT116+Ch3 cells were treated with 10 or 20μM doses of Cr[VI] for 4 hours and then assayed for a reduction in mitotic cells indicative of G2 checkpoint activation. **(B)** Comparison of Cr-induced mutation frequencies in MLH1-proficient and deficient cells exposed to 10μM doses of the Cr compounds for 24 hours (^*^ indicates p-value<.05). **(C)** Addition of Ch3 to HCT116 cells results in reduced mutation frequency among treatments with all three chromium intermediates.

The mismatch repair system plays a key role in recognizing and repairing all single base mispairs, small insertions, and deletions. One of the DNA lesions recognized by the mismatch repair system is the oxidized base adduct 8-oxoguanine [15, 16], and recent reports indicate that Cr[VI]-induced DNA oxidation results in the production of this adduct in treated cells [17, 18]. Functional MMR has also been implicated in the activation of G2/M cell cycle checkpoint in response to DNA-methylating agents [19]. This compelled us to test whether the mismatch repair system is required for Cr-induced G2/M checkpoint. The HCT116 cell line, used in the HPRT mutagenesis experiments, is known to have a homozygous mutation in the mismatch repair gene located on human chromosome 3, MLH1, and therefore is defective in mismatch repair. Introducing chromosome 3 back into HCT116 (HCT116+Ch3) restores detectable levels of MLH1 and alleviates the functional defect on mismatch repair [20]. Therefore, these two cell lines were utilized to study the role of MLH1 in Cr response. We examined the role of mismatch repair in G2/M checkpoint activation following Cr[VI] exposure by treating cells with 10 and 20μM doses of Cr[VI] for 4 hours, following which they were harvested and the G2/M checkpoint assay was performed. HCT116+Ch3 cells exhibited a marked reduction in percentage of mitotic cells following Cr[VI] exposure (Figure 4A), indicating G2 checkpoint activation. However, HCT116 cells showed only slight reduction in mitotic cells following Cr[VI] exposure, suggesting that MLH1 is involved in G2/M checkpoint regulation in response to Cr[VI] exposure.

### Mismatch repair is required to limit Cr[VI]-induced mutagenesis

The mismatch repair system is a nucleotide excision repair mechanism primarily involved in the repair of nucleotide misincorporation arising during replication [21]. Studies [17, 18] have demonstrated that Cr[VI] exposure results in oxidation of guanine to the mutagenic 8-oxoguanine adduct. Since these adducts can be recognized by the mismatch repair system, it is likely that mismatch repair system is crucial for cellular response to Cr[VI]-induced DNA damage.

Functional mismatch repair aids in limiting mutation induced by several types of genotoxins. Because MLH1 is critical for cellular response to Cr exposure, we decided to examine the role of mismatch repair in limiting Cr[VI]-induced mutation. Statistical analysis to determine the Cr[VI]-induced mutation rates relative to control revealed that Cr[VI] treatment induces a 4.35-fold (p=.0102) increase in HPRT mutation in cells lacking MLH1 vs. cells with functional MLH1, indicating that functional mismatch repair is essential for limiting Cr[VI]-induced mutation (Figure 4B). Exposure of the isogenic cell lines to different Cr compounds revealed that in an intact mismatch repair system, while Cr[V] exposure poses limited Mf in the HPRT gene, Cr[IV] remained the most genotoxic compound capable of significantly increasing Mf.

## DISCUSSION

We previously demonstrated that cellular exposure to Cr[VI] induces DNA double strand breaks [5], however, since Cr[VI] cannot directly interact with DNA, it is likely that intermediates produced during Cr[VI] metabolism are responsible for Cr[VI]-induced DNA damage. Studies involving metabolically generated chromate intermediates are limited due to instability of these compounds. Using synthetic compounds that are relatively stable, the present data have provided pivotal evidence that exposure to either Cr[IV] or Cr[V] compounds can induce DNA double-strand breaks. This indicates that both intermediates play a role in Cr[VI]-induced genotoxicity; however, human cells respond to Cr[IV] and Cr[V] differently. In response to Cr[IV]-induced DNA damage, there is no initiation of cell cycle checkpoints and no activation of the checkpoint kinase ATM. These observations are striking, given that Cr[IV] exposure results in DNA double-strand break formation. It must be noted that the synthetic Cr[V] and Cr[IV] compounds used for these experiments are stabilized by numerous oxygen substituents and it has been argued that any DNA damage caused by these compounds could be a result of oxygen radicals generated by their metabolism; however, if this were the case, the DNA damage response elicited by these compounds would likely be similar for both Cr[V] and Cr[IV]. Since our observations indicate that this is clearly not the case, we feel confident that our findings point to a possible mechanism for Cr-induced mutagenesis and carcinogenesis, although other cellular response mechanisms may be required to cope with Cr[IV]-induced DNA damage. Our data is consistent with previous findings that that Cr[VI] and Cr[V] but not Cr[IV] cause p53-independent intrinsic mitochondrial apoptosis [22, 23]. In addition, it has been also shown that Cr[IV] appears to be more efficient than Cr[VI] in producing somatic recombination in a *Drosophila* study [2].

The cellular viability experiments indicate that exposure to Cr[VI] or Cr[V] induces significant cell death, yet ten-fold greater concentrations of Cr[IV] are required to induce a similar amount of cell death compared to Cr[VI] or Cr[V]. This is surprising given the observation that all the Cr compounds exhibit equal genotoxic potential at doses as low as 10μM. These observations indicate that Cr[V] is the primary cytotoxic intermediate produced by Cr[VI] metabolism, and again point to an altered cellular response to Cr[IV]-induced genotoxicity.

Cr (VI)-containing compounds are well known mutagens and carcinogens. It was generally believed that Cr (VI)-induced cellular responses are mediated by the reactive intermediates generated directly from Cr (VI) reduction, such as reactive oxygen species (ROS) [24]. When Cr (VI)-enters the cell, it ultimately gets reduced to Cr(III), which mediates its toxicity via induction of oxidative stress during the reduction while Cr intermediates react with protein and DNA. Cr(III) can form adducts with DNA that may lead to mutations (1). Extracellular Cr(VI) iron can also induce a wide variety of DNA lesions including Cr-DNA adducts, DNA- protein crosslinks, DNA-DNA crosslinks, and oxidative damage by producing a series of reactive intermediates and ROS in cells [25, 26]. It was indicated that the induction of ROS was in a time-dependent and dose-dependent manner in the reduction of Cr(VI) by various biological systems, in particular, microsomes, mitochondria, and ascorbate [27, 28]. The oxidation-reduction system and some reduction molecules played a role in the maintenance of cellular redox balance after Cr(VI) is taken up by cells [29]. Induced ROS may further cascade multiple intracellular signaling pathways, including NF-κB, JNK/SAPK/p38, as well as Erk/MAPK. These signaling circuits can lead to transcriptional regulation of target genes that could promote proliferation or confer apoptosis resistance to exposed cells. The significance of these additional modes depends on tissue, cell-type and is often masked by alternate oncogenic mechanisms [30].

These observations led us to question whether the failure of optimal cellular response to Cr[IV]-induced DNA damage contributed to Cr[VI]-induced mutagenesis. Using the HPRT mutagenesis assay, we found Cr[IV] indeed induces more mutation than Cr[V]. Although the mechanisms resulting in this increased mutation remain unknown and are beyond the scope of this manuscript, it is reasonable that the Cr[IV] intermediate may interact with proteins involved in DNA damage response or repair. This could limit the function of these critical proteins resulting in persistence of damage and heightened mutation rates.

We also found that Cr[VI]-induced mutation is limited by functional mismatch repair. Besides its primary role in repair of mismatched bases, mismatch repair may also limit mutation through aiding in the activation of cell cycle checkpoints or apoptotic mechanisms in cells damaged by exposure to Cr[VI] [22, 23, 31]. Previous studies have linked MMR to the activation of the G2 checkpoint in response to certain alkylating agents [20, 32, 33], and ATM is thought to interact with MMR proteins to facilitate cell cycle arrest. We observed that G2 arrest in response to Cr[VI] exposure is both MMR (Figure 4A) and ATM -dependent [34]. Since ATM is also activated by exposure to Cr[VI], this provides a possible pathway required by cells for coping with DNA damage induced by Cr[VI] exposure.

In conclusion, we have demonstrated that both intermediates produced during Cr[VI] metabolism are capable of inducing DNA double strand breaks, indicating that both of these intermediates are genotoxic. However, mammalian cells do not respond properly to Cr[IV]-induced DNA damage, leading to increased mutation rates compared to Cr[V]. Although the reason human cells respond this way is still unclear, our findings support a role for tetravalent Cr[IV] as the ultimate mutagenic species generated during Cr[VI] metabolism, and point to Cr[IV] as being the Cr metabolite responsible for Cr[VI]-induced carcinogenesis.

## MATERIALS AND METHODS

### Cell culture and Cr[VI] treatment

HeLa and HCT116 cells were purchased from the American Type Culture Collection (Manassas, Va.). The HCT116 + Ch3 with reconstituted MLH1 protein expression and functional mismatch repair were described previously [12]. These cell lines were grown at 37°C in Dulbecco's Modified Eagle Medium (DMEM) supplemented with 10% fetal bovine serum in a humidified 5% CO_2_ atmosphere. Potassium Chromate (K_2_CrO_4_) was obtained from Sigma (St. Louis, MO) and was dissolved in sterile PBS. K_3_CrO_8_ (Cr[V]) and Cr(diethylenetriamine)(O_2_)_2_.H_2_O (Cr[IV]) were obtained from N.S. Dalal at Florida State University. Cr[V] was dissolved in sterile KOH and Cr[IV] was dissolved in sterile PBS prior to use.

Head and neck squamous cell carcinoma FaDu (ATCC HTB-43) cells were also used for the study, which the cells were originated from pharynx epithelia. FaDu cells were maintained in EMEM. Norma human lung fibroblast 19Lu cells were purchased from ATCC (CC1-210), and mouse lung fibroblast WT13 cells were kindly donated by Dr. Ding, Division of Pulmonary, Allergy & Critical Car Medicine at UAB. 19Lu cells were cultured in MEM and WT13 cells were in DMEM. These cells were grown at 37°C with 5% CO2 and the medium was supplemented with 10% FBS (Sigma-Aldrich) and 100 units/ml penicillin, 100μg/ml streptomycin.

### Single cell gel electrophoresis (comet assay)

The comet assay procedure used is based on the method outlined by Singh *et al* [13]. A base layer of molten 1.0% agarose was placed on microscope slides and allowed to solidify, following which, ~10^4^ treated or untreated cells were mixed with 75 μl of 1% low-melting point agarose and applied to the slide. A glass coverslip was then overlaid on the cell layer, and the agarose was allowed to solidify. The coverslip was then removed, and a third layer of low melting point agarose (75 μl) was applied to the slide. Again, a coverslip was overlaid, and the agarose was allowed to solidify. Following this, the coverslip was removed, and the slides were placed in lysis solution (10 mM Tris, pH 10.0 / 2.5 M NaCl / 100 mM EDTA / 1% Triton X-100 / 10% Me_2_SO) at 4°C for 1 h. The slides were then transferred to an electrophoresis apparatus containing a 300 mM NaOH / 1 mM EDTA solution (pH 9). The slides were left in this solution for 1 h at 4°C to promote DNA unwinding and were subsequently subjected to electric current (300 mA) for 1 hr. The slides were then removed, washed three times for 5 min in neutralizing buffer (0.4 M Tris-HCl, pH 7.5) at 4°C, and stained in a 1:10,000 dilution of Sybr Gold (Molecular Probes). Cells were photographed using a Leitz microscope equipped with epi-fluorescence optics and a SPOT CCD camera.

### Immunofluorescence microscopy

HeLa cells were cultured on sterile Falcon microscope slides and were exposed to the indicated doses of Cr. Following treatment, cells were fixed for 15 min in 2% paraformaldehyde diluted in PBS. The cells were then washed three times in PBS, permeabilized for 5 min on ice in PBS + 0.2% Triton X-100, and blocked in PBS + 1% BSA for 30 min at room temperature. The slides were incubated with anti-g-H2AX (Trevigen, Gaithersburg, MD) for 1 h at room temperature, and subsequently incubated with FITC-conjugated goat anti-rabbit secondary antibody (Jackson ImmunoResearch Laboratories) for 1 h at room temperature. Following this, cells were washed in PBS and mounted using Vectashield mounting medium containing DAPI to counter stain nuclei (Vector Laboratories). Images were captured with a Leitz epifluorescence microscope equipped with a SPOT CCD camera.

### S-phase checkpoint assay

Activation of the S-phase checkpoint was determined using the method of Garg et al [14]. HeLa cells were prelabeled for ~24 hr by culture in DMEM containing 10 nCi/mL [^14^C]-thymidine. Following prelabeling, the medium containing [^14^C]-thymidine was replaced with normal DMEM, and the cells were incubated for 6 hr. The cells were then treated with Cr for 4 hours, followed by pulse-labeling with 2.5 μCi/mL [^3^H]-thymidine for 15 min. Following pulse-labeling, cells were harvested, washed twice with PBS, and fixed in cold (-4 °C) 70% methanol. Afterwards, the cells were applied to Whatman GF/A filters which were then rinsed sequentially in 70% and 95% methanol, air dried, and then placed in glass vials containing 3 ml of scintillation fluid. The filters were then analyzed on a Beckman scintillation counter with windows set to record both ^14^C and ^3^H dpm. The measure of DNA synthesis was derived from resulting ratios of^3^H cpm to ^14^C cpm and corrected for counts resulting from channel crossover.

### Flow cytometry/G2 checkpoint assay

HeLa cells were exposed to indicated doses of Cr for 4 hours or 6 Gy of IR for 30 minutes. Cells were then harvested using trypsin, washed in PBS, and fixed in 70% ethanol. Cells were incubated with primary phospho-histone H3 antibody at 10μg/mL dilution for 3 hours at room temperature, and then with FITC-conjugated goat anti-rabbit secondary antibody at 1:30 dilution for 30 minutes at room temperature. DNA was then stained using propidium iodide and cellular florescence was determined using a FACS calibur flow cytometer [15].

### Electrophoresis and immunoblotting

At indicated times after Cr treatment, cells were harvested and subsequently lysed in TGN buffer for 15 min on ice. After lysis, cellular debris was removed by microcentrifugation and the supernatant fraction isolated and saved. Proteins were resolved by electrophoresis on 4-15% gradient SDS-PAGE gels and transferred to nitrocellulose membranes. Membranes were then probed for 2 hours with polyclonal ATM antibody to confirm equal protein loading (Bethyl Laboratories, Montgomery, TX) or polyclonal phospho-specifc ATM S1981 antibody (Rockland, Gilbertsville, PA). The membranes were then probed with HRP-conjugated secondary antibody for 1 h and the blots visualized by ECL western blotting detection reagents (Amersham Biosciences, Piscataway, NJ).

### Hypoxanthine-guanine phosphoribosyl transferase (HPRT) mutation assay

HCT116 or HCT116 + Ch3 cells were pre-selected for functional HPRT by expanding the cultures for 10 days in medium supplemented with 1x HAT (100x lyophilized HAT includes 10 mmol/L sodium hypoxanthine, 40 μmol/L aminopterin, and 1.6 mmol/L thymidine, Life Technologies, Inc.). HAT medium was then removed and the cultures were allowed to recover in normal medium for 3 days. The cells were then exposed to 10μM concentrations of our various Cr compounds for 24 hours, following which, the Cr-containing medium was removed, the cells washed with PBS, and normal growth medium added. The cells were allowed to recover for a period of 9 days, with 1.0 × 10^6^ cells being passaged every 3^rd^ day. Following this, cells were plated in triplicate on 100mm dishes at a density of 3.0 × 10^5^/dish. These cells were grown in medium containing 5mg/mL 6-TG to select for HPRT mutants. Cells treated with our Cr compounds were also plated in 100mm dishes containing no 6-TG at a density of 3.0 × 10^2^ to determine the clonal survival value after the various Cr treatments. After 10 days, the medium was removed and colonies were stained and counted. The Cr-induced mutation rate was calculated as MR^Chromium^=colony # after selection/(# cells plated × clonal survival value after chromium treatment).

### MTT assay

HeLa cellswere plated in 96 well plates at a density of 1 × 10^4^ and allowed to grow overnight. These cells were then exposed to the stated concentrations of our various chromium compounds for 24 hours, following which, 20μL of MTT reagent (5 mg/mL dissolved in PBS) was added to each well for a period of 5 hours. The medium was then removed and 100μL of DMSO was added to each well. After gentle mixing, the plates were incubated at 37°C for 5 minutes. Absorbance at 550nm was then determined using a BioRad m680 microplate reader.

## Abbreviations

Cr[VI]: Hexavalent chromium
MMR: mismatch repair
ATM: the ataxia-telangiectasia mutated
HPRT: hypoxanthine phosphoribosyltransferase
MTT: 3-(4, 5-dimethylthiazolyl-2)-2, 5-diphenyltetrazolium bromide
Mf: mutant frequency
